# Ethyl 5-methyl-1*H*-pyrrole-2-carboxyl­ate

**DOI:** 10.1107/S1600536811021180

**Published:** 2011-06-04

**Authors:** Zhao-Po Zhang, Yuan Wang, Xiao-Xia Li

**Affiliations:** a Department of Physics and Chemistry, Henan Polytechnic University, Jiaozuo 454000, People’s Republic of China; b Institute of Functional Materials, Jiangxi University of Finance and Economics, Nanchang 330013, People’s Republic of China

## Abstract

In the title mol­ecule, C_8_H_11_NO_2_, the r.m.s. deviation of non-H atoms from their best plane is 0.031 Å. Mol­ecules are connected *via* a pair of N—H⋯O hydrogen bonds into a centrosymmetric dimer.

## Related literature

For the crystal structure of the 5-cyano analogue of the title compound, see: Zhang *et al.* (2003 ▶). For the synthesis of the title compound, see: Motekaitis *et al.* (1970 ▶).
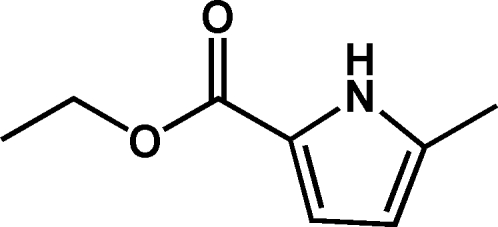


## Experimental

### 

#### Crystal data

C_8_H_11_NO_2_
*M* *_r_* = 153.18
Monoclinic, 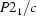

*a* = 7.0759 (3) Å
*b* = 18.0705 (9) Å
*c* = 6.6606 (3) Å
β = 101.349 (3)°
*V* = 835.01 (7) Å^3^
*Z* = 4
Mo *K*α radiation
μ = 0.09 mm^−1^
*T* = 296 K
0.20 × 0.15 × 0.13 mm

#### Data collection

Bruker APEXII CCD diffractometer
Absorption correction: multi-scan (*SADABS*; Bruker, 2007 ▶) *T*_min_ = 0.984, *T*_max_ = 0.989
7496 measured reflections
1991 independent reflections
1157 reflections with *I* > 2σ(*I*)
*R*_int_ = 0.029

#### Refinement

*R*[*F* ^2^ > 2σ(*F* ^2^)] = 0.045
*wR*(*F* ^2^) = 0.143
*S* = 1.02
1991 reflections
102 parameters
H-atom parameters constrained
Δρ_max_ = 0.16 e Å^−3^
Δρ_min_ = −0.16 e Å^−3^

### 

Data collection: *APEX2* (Bruker, 2007 ▶); cell refinement: *SAINT* (Bruker, 2007 ▶); data reduction: *SAINT*; program(s) used to solve structure: *SHELXS97* (Sheldrick, 2008 ▶); program(s) used to refine structure: *SHELXL97* (Sheldrick, 2008 ▶); molecular graphics: *SHELXTL* (Sheldrick, 2008 ▶); software used to prepare material for publication: *SHELXTL*.

## Supplementary Material

Crystal structure: contains datablock(s) I, global. DOI: 10.1107/S1600536811021180/gk2381sup1.cif

Structure factors: contains datablock(s) I. DOI: 10.1107/S1600536811021180/gk2381Isup2.hkl

Supplementary material file. DOI: 10.1107/S1600536811021180/gk2381Isup3.cml

Additional supplementary materials:  crystallographic information; 3D view; checkCIF report

## Figures and Tables

**Table 1:** Hydrogen-bond geometry (Å, °)

*D*—H⋯*A*	*D*—H	H⋯*A*	*D*⋯*A*	*D*—H⋯*A*
N1—H1⋯O1^i^	0.86	1.99	2.8363 (19)	167

Symmetry code: (i) 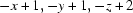.

**Fig. 1.**
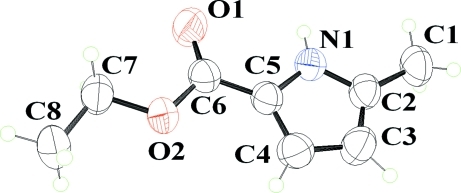
The title compound with the displacement ellipsoids shown at the 50% probability level.

**Fig. 2.**
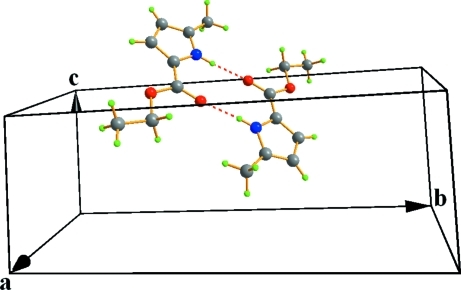
The centrosymmetric dimer via N-H···O hydrogen bonds of the title compound (hydrogen bonds shown as dashed lines, symmetry code:: 1 - x, 1 - y, 2 - z).

## supplementary crystallographic information

### Comment

As part of our studies on hydrazone ligands bearing pyrrole unit the title compound was synthesized and characterized by X-ray diffraction.

The non-hydrogen atoms of the title molecule (Fig. 1) are situated in a fair plane (r.m.s. deviation of the non-hydrogen atoms being 0.0306 Å). In the crystal, the molecules are linked into a centrosymmetric dimer by two intermolecular N—H···O hydrogen bonds, forming a *R*_2_^2^(10) ring motif (Table 1, Fig. 2).

### Experimental

The title compound was synthesized according to the literature procedure (Motekaitis *et al.*, 1970). The crude orange solid was washed well with ice water and dried in air. Colorless plates of the title compound were obtained by slow evaporation of petroleum ether/ethyl acetate (100:1) solution.

### Refinement

All H atoms were placed in calculated positions, with C—H = 0.93–0.97 Å and N—H = 0.86 Å, and were thereafter treated as riding, with *U*_iso_(H) values of 1.5Ueq(C) for methyl groups and 1.2Ueq(C,*N*) for others.

### Crystal data

**Table:**

C_8_H_11_NO_2_	*F*(000) = 328
*M**_r_* = 153.18	*D*_x_ = 1.218 Mg m^−^^3^
Monoclinic, *P*2_1_/*c*	Mo *K*α radiation, λ = 0.71073 Å
Hall symbol: -P 2ybc	Cell parameters from 2070 reflections
*a* = 7.0759 (3) Å	θ = 2.3–24.2°
*b* = 18.0705 (9) Å	µ = 0.09 mm^−^^1^
*c* = 6.6606 (3) Å	*T* = 296 K
β = 101.349 (3)°	Plate, colorless
*V* = 835.01 (7) Å^3^	0.20 × 0.15 × 0.13 mm
*Z* = 4	

### Data collection

**Table:**

Bruker APEXII CCD diffractometer	1991 independent reflections
Radiation source: fine-focus sealed tube	1157 reflections with *I* > 2σ(*I*)
graphite	*R*_int_ = 0.029
φ and ω scans	θ_max_ = 27.9°, θ_min_ = 2.3°
Absorption correction: multi-scan (*SADABS*; Bruker, 2007)	*h* = −9→9
*T*_min_ = 0.984, *T*_max_ = 0.989	*k* = −23→23
7496 measured reflections	*l* = −8→8

### Refinement

**Table:**

Refinement on *F*^2^	Primary atom site location: structure-invariant direct methods
Least-squares matrix: full	Secondary atom site location: difference Fourier map
*R*[*F*^2^ > 2σ(*F*^2^)] = 0.045	Hydrogen site location: inferred from neighbouring sites
*wR*(*F*^2^) = 0.143	H-atom parameters constrained
*S* = 1.02	*w* = 1/[σ^2^(*F*_o_^2^) + (0.0683*P*)^2^ + 0.0738*P*] where *P* = (*F*_o_^2^ + 2*F*_c_^2^)/3
1991 reflections	(Δ/σ)_max_ < 0.001
102 parameters	Δρ_max_ = 0.16 e Å^−^^3^
0 restraints	Δρ_min_ = −0.16 e Å^−^^3^

### Special details

**Table:**

Geometry. All e.s.d.'s (except the e.s.d. in the dihedral angle between two l.s. planes) are estimated using the full covariance matrix. The cell e.s.d.'s are taken into account individually in the estimation of e.s.d.'s in distances, angles and torsion angles; correlations between e.s.d.'s in cell parameters are only used when they are defined by crystal symmetry. An approximate (isotropic) treatment of cell e.s.d.'s is used for estimating e.s.d.'s involving l.s. planes.
Refinement. Refinement of *F*^2^ against ALL reflections. The weighted *R*-factor *wR* and goodness of fit *S* are based on *F*^2^, conventional *R*-factors *R* are based on *F*, with *F* set to zero for negative *F*^2^. The threshold expression of *F*^2^ > σ(*F*^2^) is used only for calculating *R*-factors(gt) *etc*. and is not relevant to the choice of reflections for refinement. *R*-factors based on *F*^2^ are statistically about twice as large as those based on *F*, and *R*- factors based on ALL data will be even larger.

### Fractional atomic coordinates and isotropic or equivalent isotropic displacement parameters (Å^2^)

**Table:**

	*x*	*y*	*z*	*U*_iso_*/*U*_eq_	
O2	0.12507 (16)	0.64959 (6)	0.92921 (18)	0.0629 (4)	
N1	0.52408 (19)	0.57393 (7)	0.7402 (2)	0.0581 (4)	
H1	0.5637	0.5360	0.8146	0.087*	
C6	0.2631 (2)	0.60036 (9)	0.9187 (3)	0.0585 (4)	
O1	0.2888 (2)	0.54602 (8)	1.0272 (2)	0.0861 (5)	
C5	0.3729 (2)	0.61798 (9)	0.7644 (2)	0.0548 (4)	
C2	0.6023 (2)	0.59835 (10)	0.5831 (3)	0.0600 (5)	
C8	−0.1423 (3)	0.69326 (11)	1.0591 (3)	0.0768 (6)	
H8A	−0.0794	0.7400	1.0916	0.115*	
H8B	−0.2268	0.6837	1.1527	0.115*	
H8C	−0.2159	0.6946	0.9217	0.115*	
C4	0.3561 (2)	0.67196 (10)	0.6178 (3)	0.0651 (5)	
H4	0.2658	0.7100	0.5976	0.078*	
C7	0.0051 (3)	0.63353 (10)	1.0768 (3)	0.0691 (5)	
H7A	0.0825	0.6324	1.2142	0.083*	
H7B	−0.0569	0.5858	1.0480	0.083*	
C1	0.7719 (3)	0.56133 (11)	0.5261 (3)	0.0780 (6)	
H1A	0.8862	0.5893	0.5791	0.117*	
H1B	0.7543	0.5585	0.3796	0.117*	
H1C	0.7847	0.5123	0.5827	0.117*	
C3	0.4987 (3)	0.65956 (10)	0.5044 (3)	0.0712 (5)	
H3	0.5200	0.6879	0.3944	0.085*	

### Atomic displacement parameters (Å^2^)

**Table:**

	*U*^11^	*U*^22^	*U*^33^	*U*^12^	*U*^13^	*U*^23^
O2	0.0581 (7)	0.0624 (7)	0.0748 (8)	0.0073 (5)	0.0294 (6)	0.0035 (6)
N1	0.0533 (8)	0.0560 (8)	0.0691 (9)	0.0001 (6)	0.0221 (7)	0.0000 (6)
C6	0.0519 (9)	0.0563 (9)	0.0698 (11)	0.0019 (8)	0.0182 (8)	−0.0026 (9)
O1	0.0847 (10)	0.0820 (9)	0.1032 (11)	0.0273 (8)	0.0467 (8)	0.0332 (8)
C5	0.0486 (9)	0.0532 (9)	0.0649 (11)	−0.0015 (7)	0.0165 (8)	−0.0035 (8)
C2	0.0539 (9)	0.0652 (10)	0.0644 (11)	−0.0091 (8)	0.0202 (8)	−0.0063 (8)
C8	0.0689 (12)	0.0819 (13)	0.0884 (15)	0.0074 (10)	0.0373 (11)	−0.0054 (10)
C4	0.0587 (10)	0.0616 (10)	0.0766 (12)	0.0036 (8)	0.0172 (9)	0.0060 (9)
C7	0.0635 (11)	0.0788 (12)	0.0716 (12)	0.0050 (9)	0.0293 (9)	0.0048 (9)
C1	0.0664 (12)	0.0894 (14)	0.0867 (14)	−0.0022 (10)	0.0360 (10)	−0.0094 (11)
C3	0.0683 (11)	0.0781 (12)	0.0715 (12)	−0.0023 (10)	0.0246 (10)	0.0107 (10)

### Geometric parameters (Å, °)

**Table:**

C2—C1	1.487 (2)	C1—H1A	0.9600
C2—C3	1.373 (3)	C1—H1B	0.9600
C4—C3	1.392 (2)	C1—H1C	0.9600
C5—C4	1.369 (2)	C3—H3	0.9300
N1—C2	1.351 (2)	C4—H4	0.9300
N1—C5	1.368 (2)	C7—H7A	0.9700
C6—C5	1.440 (2)	C7—H7B	0.9700
C6—O1	1.212 (2)	C8—H8A	0.9600
O2—C6	1.3333 (19)	C8—H8B	0.9600
O2—C7	1.4490 (19)	C8—H8C	0.9600
C8—C7	1.490 (2)	N1—H1	0.8600
			
C6—O2—C7	115.81 (13)	C5—C4—C3	107.57 (16)
C2—N1—C5	110.53 (14)	C5—C4—H4	126.2
C2—N1—H1	124.7	C3—C4—H4	126.2
C5—N1—H1	124.7	O2—C7—C8	107.22 (15)
O1—C6—O2	122.31 (15)	O2—C7—H7A	110.3
O1—C6—C5	124.50 (15)	C8—C7—H7A	110.3
O2—C6—C5	113.19 (15)	O2—C7—H7B	110.3
C4—C5—N1	106.89 (14)	C8—C7—H7B	110.3
C4—C5—C6	133.09 (16)	H7A—C7—H7B	108.5
N1—C5—C6	119.97 (15)	C2—C1—H1A	109.5
N1—C2—C3	106.81 (15)	C2—C1—H1B	109.5
N1—C2—C1	121.69 (16)	H1A—C1—H1B	109.5
C3—C2—C1	131.48 (17)	C2—C1—H1C	109.5
C7—C8—H8A	109.5	H1A—C1—H1C	109.5
C7—C8—H8B	109.5	H1B—C1—H1C	109.5
H8A—C8—H8B	109.5	C2—C3—C4	108.20 (16)
C7—C8—H8C	109.5	C2—C3—H3	125.9
H8A—C8—H8C	109.5	C4—C3—H3	125.9
H8B—C8—H8C	109.5		
			
C7—O2—C6—O1	−0.9 (2)	C5—N1—C2—C3	−0.1 (2)
C7—O2—C6—C5	177.93 (14)	C5—N1—C2—C1	178.73 (15)
C2—N1—C5—C4	−0.12 (19)	N1—C5—C4—C3	0.26 (19)
C2—N1—C5—C6	177.58 (14)	C6—C5—C4—C3	−177.02 (18)
O1—C6—C5—C4	173.57 (18)	C6—O2—C7—C8	−178.10 (14)
O2—C6—C5—C4	−5.3 (3)	N1—C2—C3—C4	0.2 (2)
O1—C6—C5—N1	−3.4 (3)	C1—C2—C3—C4	−178.40 (18)
O2—C6—C5—N1	177.75 (13)	C5—C4—C3—C2	−0.3 (2)

### Hydrogen-bond geometry (Å, °)

**Table:**

*D*—H···*A*	*D*—H	H···*A*	*D*···*A*	*D*—H···*A*
N1—H1···O1^i^	0.86	1.99	2.8363 (19)	167.

Symmetry codes: (i) −*x*+1, −*y*+1, −*z*+2.

## References

[bb1] Bruker (2007). *APEX2*, *SAINT* and *SADABS* Bruker AXS Inc., Madison, Wisconsin, USA.

[bb2] Motekaitis, R. J., Heinert, H. D. & Martell, A. E. (1970). *J. Org. Chem.* **35**, 2504–2511.

[bb3] Sheldrick, G. M. (2008). *Acta Cryst.* A**64**, 112–122. 10.1107/S0108767307043930 18156677

[bb4] Zhang, Y.-H., Yin, Z., Li, X.-F., He, J. & Cheng, J.-P. (2003). *Acta Cryst.* E**59**, o1881–o1882.

